# Development and Characterization of Monoclonal Antibodies Against VP3 Protein of Infectious Bursal Disease Virus

**DOI:** 10.1155/tbed/5915042

**Published:** 2025-11-21

**Authors:** Xiao-Ya Pan, Haojie Ren, Meng-Hui Zi, Jun-Hao Fan, Yu-He Ma, Han-Cheng Shao, Zhi-Shan Liang, Yuhang Zhang, Shichong Han, Gai-Ping Zhang, Bo Wan, Wencheng Lin, Wen-Rui He

**Affiliations:** ^1^International Joint Research Centre of National Animal Immunology, College of Veterinary Medicine, Henan Agricultural University, Zhengzhou, Henan, China; ^2^College of Animal Science, South China Agricultural University, Guangzhou, China; ^3^Longhu Laboratory, Zhengzhou, Henan, China; ^4^Ministry of Education Key Laboratory for Animal Pathogens and Biosafety, Henan Agricultural University, Zhengzhou, China

**Keywords:** antigenic epitope mapping, infectious bursal disease virus, monoclonal antibodies, VP3

## Abstract

Infectious bursal disease virus (IBDV) causes an acute, highly contagious and immunosuppressive disease in 3–5-week-old chicken, called infectious bursal disease (IBD). Current vaccines targeting the hypervariable VP2 gene fail to provide cross-protection against different IBDV strains, necessitating the development of novel diagnostic and preventive strategies that explore other candidate genes to ensure immune efficacy. Here, VP3, a conserved nucleocapsid protein of IBDV, was selected for further analysis. A prokaryotic expression vector, pET-32a-IBDV-VP3, was constructed, followed by expression and purification of the recombinant protein. Following the intraperitoneal injection of recombinant proteins into the mice, eight monoclonal antibodies (mAbs) were identified by hybridoma cell fusion, clone purification, and immunological assays. Among the mAbs, mAb 19D8 effectively neutralized IBDV infection during viral attachment and penetration. Antigenic epitopes of mAb 19D8 were identified using alanine-scanning mutagenesis. Our results showed that four amino acids, F20, K21, T23, and E25, located on an α-helix of the VP3, were the key amino acids recognized by 19D8. Homologous and structural analyses revealed that these sites were highly conserved across different IBDV strains from diverse regions. These findings provide crucial insights into the antigenicity of VP3 and underscore the potential of VP3 as a target for the development of broad-spectrum diagnostic tools and cross-protection vaccines against IBDV.

## 1. Introduction

The infectious bursal disease virus (IBDV) is a small, nonenveloped RNA virus with an icosahedral nucleocapsid structure. The virus is remarkably stable and persists in the environment despite extensive cleaning and disinfection efforts [1]. With regard to clinical infections caused by IBDV, domestic chickens serve as the primary avian host, and the virus targets immature B lymphocytes in the bursa of Fabricius. Infected chicks with low levels of neutralizing antibodies or compromised immune systems show increased mortality [2–5]. Research has demonstrated that IBDV infection induces profound immunosuppression in chickens, significantly increases their susceptibility to secondary infections by opportunistic pathogens [6]. Despite extensive immunization efforts, outbreaks persist due to the emergence of antigenically divergent strains and limitations of cross-protective immunity, underscoring the need for improved diagnostic and therapeutic tools.

IBDV has a double-stranded RNA genome encoding two segments, A and B. Segment A (3.17 kb) has two partially overlapping open reading frames (ORF) [6–11], in which ORF A1 encodes the nonstructural viral protein VP5 [12] and ORF A2 encodes a large polyprotein that undergoes cleavage by the protease VP4 to yield the proteins VP2, VP4, and VP3 [13–15]. VP2 and VP3 are the primary structural proteins, accounting for 51% and 40% of the virions, respectively [16]. Segment B (2.8 kb) contains an ORF that encodes VP1, an RNA-dependent RNA polymerase enzyme [17]. VP3 is a multifunctional structural protein that is critical for viral replication and assembly [18]. It forms the inner capsid scaffold [19], interacts with the viral RNA-dependent RNA polymerase (VP1), and plays a role in evading host immune responses by binding to double-stranded RNA [20]. Although VP2 exhibits greater immunogenicity in inducing neutralizing antibodies [21], the VP3 antigenic epitope is remarkable for its conservation, stability, cross-protection capabilities, and cellular immune activation. The integration of antigenic epitopes from both VP2 and VP3 may achieve a more comprehensive and robust protective effect in vaccine development. Thus, the conserved nature of VP3 across IBDV strains makes it an attractive candidate for the development of broad-spectrum diagnostic reagents and therapeutic interventions.

Monoclonal antibodies (mAbs) have emerged as an indispensable tool in virology for diagnostics, pathogenesis studies, and therapeutic applications owing to their high specificity, reproducibility, and ease of commercial production. mAbs targeting conserved VP3 antigenic epitopes not only complement VP2-based diagnostic approaches but also broaden strain coverage and enhance our understanding of viral neutralization mechanisms. The current focus is on neutralizing antibodies against VP2, and the immunogenic potential of VP3 remains underexplored [22]. Previous research has primarily examined the role of VP3 in viral replication; however, its surface-exposed antigenic epitopes offer promising opportunities for immune intervention. To date, only a limited number of VP3-specific mAbs have been identified, and the neutralizing capabilities and antigenic epitope characteristics of those mAbs are yet to be fully defined.

In this study, we describe the development and characterization of murine mAbs targeting the VP3 protein of IBDV. A combination of hybridoma screening, structural modeling, and functional analysis led to the identification of a neutralizing mAb (19D8) that binds to a conserved antigenic epitope on VP3. Our results provide an alternative to immunization vaccines centered on VP2. This study enhances the understanding of the antigenic structure of VP3 and offers practical tools for improving IBDV surveillance and vaccine development.

## 2. Materials and Methods

### 2.1. Cells and Regents

The HEK293T cells were kindly provided by Prof. Hong-Bing Shu. They were cultured in Dulbecco's modified Eagle's medium (Solarbio, Beijing, China) supplemented with 10% fetal bovine serum (FBS, Gibco/Thermo Fisher Scientific, Waltham, MA, USA) at 37°C in a 5% CO_2_ atmosphere. DF-1 and HD11 cells were acquired from the American Type Culture Collection (ATCC, Manassas, VA, USA). DF-1 cells were cultured in Dulbecco's modified Eagle medium, whereas HD11 cells were maintained in RPMI 1640 medium (Solarbio, Beijing, China). Chicken embryo fibroblast (CEF) cells are isolated from chicken embryos aged 9–11 days. The chicken embryos were purchased from Boehringer Ingelheim (Beijing, China). CEF cells were maintained in DMEM/F12 medium (Thermo Fisher Scientific Inc., USA). All culture media were supplemented with 10% FBS, and all cultures were incubated in a humidified atmosphere containing 5% CO_2_. Mouse myeloma cells (SP2/0) were obtained from the ATCC and cultured in hybridoma cell serum-free medium (Basal Media, Shanghai, China) at 37°C with 5% CO_2_. Lx, a cell culture-adapted IBDV strain, was provided by Prof. Jue Liu from Yangzhou University. Competent cells of *E. coli* strains DH5*α* and DE3 were purchased from Tsingke Biotechnology (Beijing, China). The mouse IgG (A7028) was sourced from Beyotime. The chicken anti-IBDV positive serum was generously provided by Professor Dawei Jiang from Henan Agricultural University.

### 2.2. Constructs

Based on the VP3 sequence of the IBDV Lx strain from the National Center for Biotechnology Information (NCBI) database, specific primers were designed by conjugating *EcoRI* and *XhoI* sites (Table 1). The target gene was amplified using a laboratory-preserved pRK-VP3 plasmid as a template. Next, the target gene was fused to the pET-32a vector using a homologous recombinase (Vazyme, Nanjing, China) at the restriction sites. The homologous recombination system is presented in Table 2. After PCR and restriction enzyme *EcoRI* (NEB, R0101S, USA) and *XhoI* (NEB, R0146S, USA) double digestion, the correct construct was confirmed by DNA sequencing (Shenggong, Shanghai, China). The pET-32a-IBDV-VP3 plasmids were successfully constructed.

### 2.3. Prokaryotic Expression and Purification of VP3

To prepare recombinant *E*. *coli* for VP3 protein expression, recombinant pET-32a plasmids were transformed into the *E. coli* BL21 (DE3) cells; IPTG was added to a final concentration of 1 mM and cells were incubated for an additional 6 h at 37°C with shaking. The enriched bacterial solution was resuspended in phosphate-buffered saline (PBS), lysed using a high-pressure homogeneous sterilizer, and subsequently centrifuged at 8000 rpm for 20 min. The expression of the VP3 protein was analyzed by sodium dodecyl sulfate–polyacrylamide gel electrophoresis (SDS-PAGE) to compare its distribution in the supernatant and precipitate fractions. The specificity of the recombinant protein was verified by Western blotting using an anti-His mAb (Proteintech, 66005-1-Ig, China). Finally, the purified VP3 protein was subsequently obtained via Ni-affinity chromatography.

### 2.4. Preparation of mAbs against VP3

6–8 weeks female BALB/c mice were immunized with 50 μg of VP3 protein antigen emulsified in an equal volume of Freund's complete adjuvant (Sigma F5881, USA) via multiple subcutaneous injections at different sites. Mice received booster immunizations every 2 weeks with the antigen emulsified in Freund's incomplete adjuvant. Following the third immunization, mouse serum samples were serially diluted in PBS at the following ratios: 1:200, 1:400, 1:800, 1:1600, 1:3200, 1:6400, and 1:12800, and serum antibody titers were quantified by ELISA. Simultaneously, Western blotting analysis was utilized to detect the serum reactivity of mice immunized with the eukaryotic VP3 protein.

An intraperitoneal injection of 50 μg of VP3 protein was administered to mice exhibiting high serum titers for booster immunization. Mice were anesthetized with 2%–5% isoflurane (20 s, intranasal) and euthanized by cervical dislocation. Spleen cells were harvested, and carcasses were sterilized by autoclaving. Subsequently, spleen cells were fused with SP2/0 myeloma cells using 50% (w/v) polyethylene glycol 1420 (PEG 1420; Sigma, 25322-68-3, USA). The resulting hybrid cells were selected in HAT medium (Sigma, H0262, USA) for 7 days in 96-well plates, followed by culture in HT medium (Sigma, H0137, USA) medium. Positive hybridoma clones identified by indirect ELISA were subjected to limited dilution subcloning for monoclonal isolation. Antibody isotypes were determined using the mouse monoclonal antibody isotyping ELISA kit (Proteintech, PK20002, China) following the manufacturer's protocol. The specificity and reactivity of the resulting mAbs were characterized by Western blotting and immunofluorescence assays (IFA).

### 2.5. Indirect ELISA

The 96-well ELISA plates were coated at 4°C with 200 ng of His-VP3 recombinant protein per well for 20 h. After washing twice with PBS, the plates were blocked with 1.5% bovine serum albumin (BSA; Solarbio, Beijing, China) in PBS at 37°C for 2 h. The cell supernatant or diluted IBDV-positive serum was added to the plate and incubated at 37°C for 1 h. The positive serum was generated by immunizing mice with purified His-VP3 protein. After washing four times with PBS containing 0.05% Tween-20 (PBST), the plate was incubated with horseradish peroxidase (HRP)-conjugated goat anti-mouse IgG (Proteintech, SA00001-1, China) at 37°C for 1 h. After washing five times, 100 μL TMB substrate (Solarbio, PR1200, China) was added to each well and incubated for 10 min at room temperature. The reaction was terminated by adding 2 M H_2_SO_4_. Optical density (OD) at 450 nm was measured using a Scientific Fluoroskan microplate reader (Thermo Fisher, USA) to evaluate antibody–antigen interactions. To ensure experimental reliability, each sample was tested in triplicate, and the final results were calculated as the mean OD_450 nm_ value from the three replicates.

### 2.6. Western Blotting

The protein samples were separated by SDS-PAGE using 12.5% or 5% polyacrylamide gels, then the proteins were transferred to polyvinylidene difluoride (PVDF) membranes by transfer buffer electrophoresis. The membranes were blocked with 5% skim milk in TBS for 1 h at 37°C, followed by incubation with postimmunization mouse serum (1:1000 dilution) or cell supernatant in 5% skim milk for 1 h at 37°C. After washing, membranes were incubated with HRP-goat anti-mouse IgG secondary antibody (Proteintech, SA00001–5, China; 1:3000) in 5% skim milk for 1 h at room temperature. Finally, protein bands were visualized using an electrochemiluminescence (ECL) detection system.

### 2.7. IFA

To assess antibody reactivity against the IBDV Lx strain, IFA was performed using DF-1 cells infected with IBDV. At 12–24 h postinfection (hpi), the cells were fixed with 4% paraformaldehyde (Biosharp, Beijing, China) for 15 min and subsequently incubated with 0.1% Triton X-100 (Solarbio, Beijing, China) to mediate cell permeabilization for 15 min. After blocking with 5% BSA for 1 h, the cells were incubated with the mouse serum antibodies or hybridoma-derived supernatants for 1 h at 37°C, followed by five washes with PBST, the cells were incubated with Dylight 488-conjugated goat anti-mouse IgG (Abbkine, A23210, China) for 1 h at 37°C. After staining with DAPI (Solarbio, C0065, China), imaging of the cells was performed using a fluorescence microscope (Olympus, Japan).

### 2.8. Neutralization Assay

First, IBDV (200 TCID_50_) was coincubated with various antibodies (0.1 mg/mL) at 37°C for 1 h to facilitate complete antibody-virus binding. Subsequently, the nonneutralized viruses were inoculated onto primary chicken embryonic fibroblasts (CEFs) and cultured for an additional 36 h. The presence of nonneutralized viruses was quantified by observing cytopathic effects (CPE) under bright-field microscopy and detecting viral protein expression through IFA.

### 2.9. Virus Adsorption and Internalization Assay

Initially, IBDV (MOI = 10) was incubated with monoclonal antibody 19D8 (0.1 mg/mL) at 37°C for 1 h to facilitate antibody–virus complex formation. Nonspecific IgG was used as the negative control group. The virus–antibody mixture was then introduced into the HD11 cell cultures and incubated at 4°C for 2 h to allow viral adsorption without internalization. Unbound viral particles were subsequently removed by washing with ice-cold PBS. At 4°C, viruses can attach to cells, but membrane fusion does not occur [23]. Total RNA was extracted from the lysed cells and reverse-transcribed for subsequent quantitative PCR (qPCR) analysis. Concurrently, cells were harvested for protein extraction to quantify adsorbed IBDV virions levels.

The aforementioned procedure was repeated. Following the final wash of unbound virus particles with ice-cold PBS, the cells were transferred to a 37°C culture environment and incubated for 2 h to facilitate viral internalization. Under these conditions, the virus particles adsorbed onto the cell membrane were internalized. Surface-bound viral particles were subsequently removed by trypsin treatment. Total RNA was extracted from the cells for qPCR analysis, and protein levels were quantified by Western blotting.

### 2.10. Reverse Transcription-Quantitative PCR (RT-qPCR)

Total RNA was extracted using the TRIzol (TaKaRa Bio, Beijing, China) reagent and reverse-transcribed into cDNA employing the HiScript III 1st Strand cDNA Synthesis Kit (Vazyme, Nanjing, China) according to the manufacturer's protocols. The resulting cDNA was served as a template for qPCR analysis. PCR reaction mixtures consisted of 0.5 μL of each gene-specific primer, 5 μL of cDNA template diluted with RNase-free water, and 5 μL of 2 × Universal SYBR Green Fast qPCR Mix. Each sample was set up in triplicate, and the expression of each gene was averaged with the corresponding internal reference and calculated by the method of 2^−ΔΔCt^. The data correspond to the relative expression levels of the specified mRNAs, which were normalized to the expression level of actin. Primers used for RT-qPCR are listed in Table 3. The data were shown as the means from representative experiments in triplicate.

### 2.11. Identification of the Antigenic Epitopes

Alanine scanning mutagenesis was employed to identify the critical amino acid residues in VP3 that are specifically recognized by the mAb 19D8. To incorporate the mutation of 6 consecutive amino acids into an alanine as a group, special primers were designed to construct the eukaryotic expression plasmids of the VP3 mutants (Table 4). Through preliminary screening, individual candidate amino acids within these regions were subsequently mutated to alanine for detailed identification (Table 5). Western blotting was employed to analyze the binding activities of the mAbs and delineating the precise antigenic epitopes of VP3.

### 2.12. Bioinformatics Analysis of VP3

*VP3* gene sequences from geographically diverse locations were retrieved from the NCBI database to assess the conservation of IBDV antigenic epitopes. Using the MEGA7 software, we systematically compared the VP3 antigenic epitopes identified by mAb 19D8 across different viral strains.

To elucidate the properties of antigenic epitopes conserved across IBDV genotypes, I-TASSER, which can be found at https://seq2fun.dcmb.med.umich.edu//I-TASSER/, was employed to predict the three-dimensional structure of the full-length IBDV VP3-protein. This computational modeling approach aimed to spatially map the conserved antigenic epitopes on the IBDV VP3 proteins. The I-TASSER algorithm selects highly threaded templates from the Protein Data Bank (PDB) for structure prediction.

### 2.13. Statistical Analysis

The GraphPad Prism software version 8.0 (La Jolla, CA, USA) was used for statistical analysis. The quantitative data were presented in histograms as mean ± standard deviation (SD). The unpaired Student's test was used for data analysis. “ns” indicates no significant difference, with *p* > 0.05. Statistical significance was set at *p*  < 0.05. Asterisks in the figures indicate statistical significance: *⁣*^*∗*^*p*  < 0.05; *⁣*^*∗∗*^*p*  < 0.01; *⁣*^*∗∗∗*^*p*  < 0.001.

## 3. Results

### 3.1. Prokaryotic Expression and Purification of VP3

**Figure 1:**
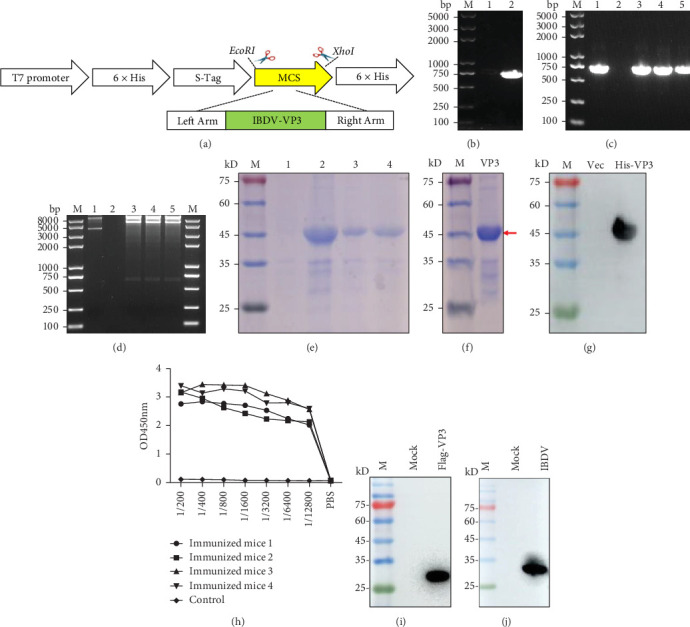
Prokaryotic expression and purification of VP3. (A) Schematic diagram illustrating the construction of the prokaryotic expression plasmid pET-32a-IBDV-VP3 via homologous recombination. (B) Amplification of the *IBDV VP3* gene. Lane 1: Negative control; Lane 2: the *VP3* gene. M: DNA marker. (C) PCR identification of the recombinant pET-32a-IBDV-VP3 prokaryotic expression plasmids. Lane 1: Positive control; Lane 2: Negative control; Lane 3–5:3 colonies to be identified. M: DNA marker. (D) Restricted enzyme digestion of the recombinant pET-32a-IBDV-VP3 prokaryotic expression plasmids. Lane 1: Negative control; Lane 2: Blank control; Lane 3–5:3 colonies of the recombinant plasmids pET-32a-IBDV-VP3. M: DNA marker. (E) Expression of the recombinant VP3 protein. Lane 1: Recombinant *Escherichia coli* DE3 without IPTG treatment; Lane 2: Recombinant DE3 with IPTG treatment; Lane 3: Supernatant of the recombinant *Escherichia coli* DE3 with IPTG treatment; Lane 4: Precipitate of the recombinant *Escherichia coli* DE3 with IPTG treatment. M: Protein molecular weight marker. (F) Purification of the recombinant VP3 protein. M: Protein molecular weight marker. (G) Identification of the recombinant VP3 protein using anti-His mAb. M: Protein molecular weight marker. (H) Serum antibody titers of mice immunized with VP3 protein were determined by indirect ELISA. (I) Detection of the eukaryotically expressed VP3 recombinant protein using mouse #3 serum. M: Protein molecular weight marker. (J) Detection of VP3 protein in DF-1 cells infected with IBDV using mouse #3 serum. M: Protein molecular weight marker.

The pET-32a-IBDV-VP3 prokaryotic expression plasmid was constructed using the homologous recombination technique (Figure 1A). Plasmids stored in the laboratory were used as templates for amplifying the target gene. The amplified fragment was approximately 705 bp long (Figure 1B). PCR amplification and restriction enzyme digestion assayses confirmed the successful cloning of the VP3 gene fragment into the pET-32a vector (Figure 1C,D). Next, pET-32a-IBDV-VP3 was transformed into *E*. c*oli* DE3, and the recombinant His-VP3 protein was detected in both the supernatant and inclusion bodies (Figure 1E). Therefore, the pET-32a-IBDV-VP3 plasmids were successfully constructed and validated for subsequent experiments.

Following purification, the recombinant His-VP3 protein was successfully obtained, exhibiting an approximate molecular weight of 45 kDa (Figure 1F). Western blotting demonstrated that the purified His-VP3 protein was recognized by anti-His mAb (Figure 1G). These results indicated that the specificity and antigenicity of the prepared His-VP3 protein met the requirements of subsequent experiments.

### 3.2. Preparation and Identification of mAbs Against VP3

After immunizing mice with the recombinant protein, the serum titer was detected by ELISA, and the titer was observed to be above 1/12800, and the antibody titers of mouse #3 was relatively higher (Figure 1H). Next, the binding capacity of serum from mouse #3 to eukaryotically expressed VP3 was evaluated. Western blotting analysis demonstrated that the antiserum obtained from immunized mouse #3 was capable of effectively recognizing the eukaryotically expressed Flag-VP3 protein (Figure 1I). Meanwhile, the antiserum from mouse #3 effectively recognized the VP3 protein expressed in virus-infected DF-1 cells (Figure 1J). These results demonstrate that immunized mouse #3 is suitable for the subsequent preparation of mAbs.

**Figure 2:**
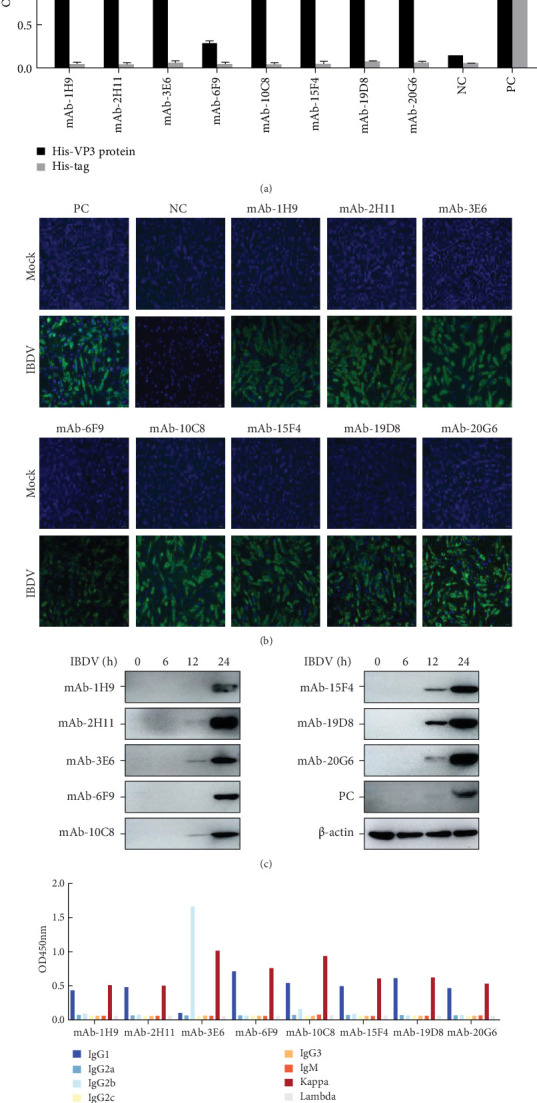
Preparation and identification of mAbs against VP3. (A) Determination of monoclonal antibody titer using the indirect ELISA method. (B) The analyzation of the mAb reactivities by IFA. Mock represents unexposed DF-1 cells. (C) Reactivity of mAbs to IBDV VP3 was analyzed by Western blotting. (D) Identification of the subtypes of anti-VP3 mAbs. Monoclonal antibody subclasses were identified using indirect ELISA, following the manufacturer's instructions for the monoclonal antibody subclass identification kit.

Through the application of hybridoma cell fusion, clone purification, and ELISA, eight mAbs capable of specifically recognizing IBDV VP3 were successfully screened and isolated. These antibodies were designated as 1H9, 2H11, 3E6, 6F9, 10C8, 15F4, 19D8, and 20G6, respectively (Figure 2A). IFA indicated that the mAbs could specifically recognize the eukaryotically expressed VP3 protein, and mAbs 19D8 and 20G6 had relatively high sensitivity (Figure 2B). The ability of the mAbs to recognize viral proteins was assessed by Western blotting. Consistently, all eight mAbs recognized and bound to IBDV (Figure 2C). Next, the subtypes of the eight mAbs were determined using the mouse monoclonal antibody isotyping ELISA kit. The results revealed that seven mAbs (1H9, 2H11, 6F9, 10C8, 15F4, 19D8, and 20G6) exhibited an IgG1 heavy chain subtype, whereas mAb 3E6 displayed an IgG2b heavy chain subtype. Notably, all eight mAbs possessed a kappa light chain (Figure 2D).

### 3.3. mAb 19D8 Effectively Neutralizes IBDV Infection During Viral Adsorption and Internalization

**Figure 3:**
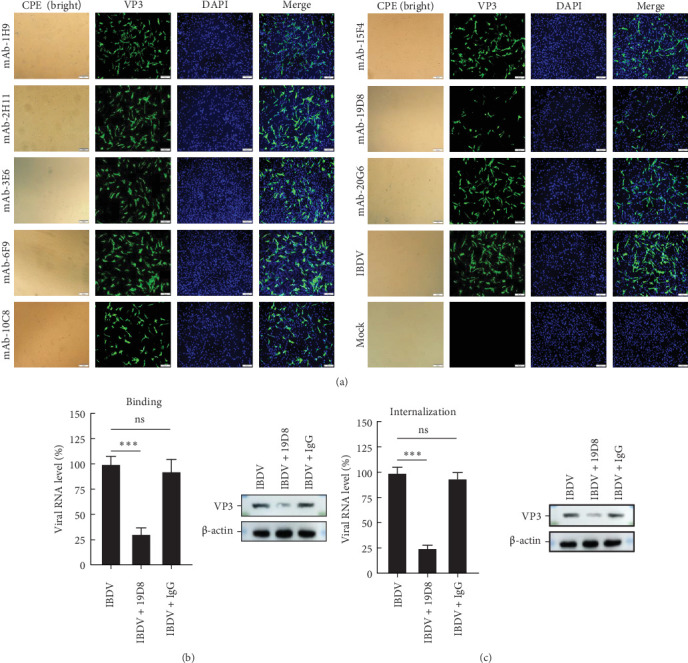
mAb 19D8 effectively neutralizes IBDV infection during viral adsorption and internalization. (A) IFA was used to determine the neutralization of the eight antibodies. (B) RT-qPCR and Western blotting analysis were employed to evaluate the impact of mAbs on the virion adsorption process. (C) RT-qPCR and Western blotting analysis were employed to evaluate the impact of mAbs on the virion internalization process. The data were shown as the means ± SDs from one representative experiment performed in triplicates. *⁣*^*∗*^*p* < 0.05; *⁣*^*∗∗*^*p* < 0.01; *⁣*^*∗∗∗*^*p* < 0.001; ns, not significant (*p* > 0.05) (unpaired Student's *t* test).

All the eight VP3-specific mAbs showed robust reactivity with recombinant VP3; however, their potential to neutralize IBDV infection remains unexplored. To assess the functional relevance of these antibodies, we performed in vitro neutralization assays using cell culture-adapted IBDV strains. Only mAb 19D8, but not the other seven mAbs, exhibited significant neutralizing activity against the IBDV Lx strain, as demonstrated by comparing the CPE and immunofluorescence (Figure 3A).

Given the neutralizing effect of mAb 19D8, its neutralization mechanism was further investigated. The initial stages of viral replication include adsorption, internalization, and membrane fusion. To ascertain the precise stage at which antibodies neutralize early viral replication, IBDV-infected HD11 cells (MOI = 10) were utilized for the virus adsorption assay. The findings demonstrated that treatment with mAb 19D8 significantly reduced virion RNA adsorption to the cell membrane compared to the control group. Western blotting analysis further confirmed that treatment with the monoclonal antibody 19D8 significantly reduced viral protein VP3 levels compared to the control group (Figure 3B). Besides, the internalization assay validated that virion RNA and protein VP3 under 19D8 treatment were dramatically lower than the controls (Figure 3C). These results indicate that the neutralization mechanism of mAb 19D8 may involve the inhibition of viral adsorption and internalization to host cells.

### 3.4. F20, K21, T23, and E25 Are the Key Amino Acids Recognized by mAb 19D8

**Figure 4:**
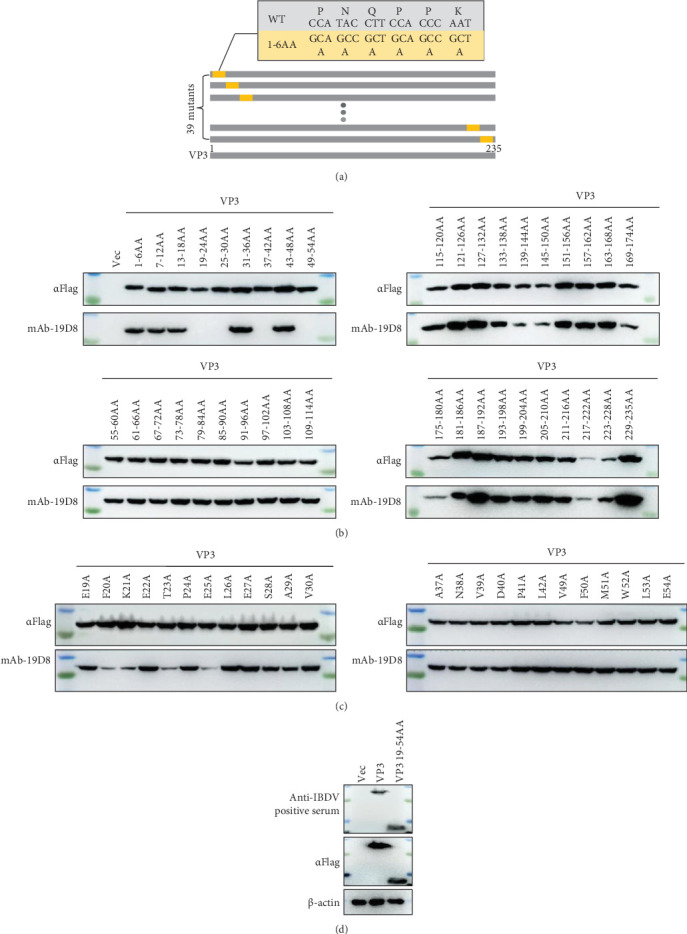
Identification of the antigenic epitope of the VP3 protein. (A) Schematic diagram of a VP3 mutant for antigenic epitope localization. (B) Preliminary mapping of the antigenic epitopes of VP3. (C) Determination of the key amino acids that mAbs recognized. (D) The reactivity of the chicken anti-IBDV positive serum with the epitope VP3 19-54AA was confirmed. AA, amino acid.

To identify the antigenic epitopes recognized by the mAbs, the expression of these mutants was detected by constructing eukaryotic VP3 protein granules containing 39 six-amino acid group mutants (Figure 4A). Through initial identification, we found that the antigenic epitope regions recognized by mAb 19D8 included 19-24AA, 25-30AA, 37-42AA, and 49-54AA (Figure 4B). Then, the antigenic epitope regions were mutated in a single amino acid, and it was determined that the key amino acids in the antigenic epitope recognized by mAb 19D8 were F20, K21, T23 and E25 (Figure 4C). The reactivity of the chicken anti-IBDV positive serum with the epitope VP3 19-54AA was confirmed by Western blotting analysis. Results demonstrated that both the truncated VP3 protein and the full-length VP3 were recognized by the anti-IBDV positive serum (Figure 4D). These findings suggest that the identified VP3 epitopes function as antigenic determinants of IBDV in chickens and are capable of eliciting a humoral immune response following IBDV infection.

**Figure 5:**
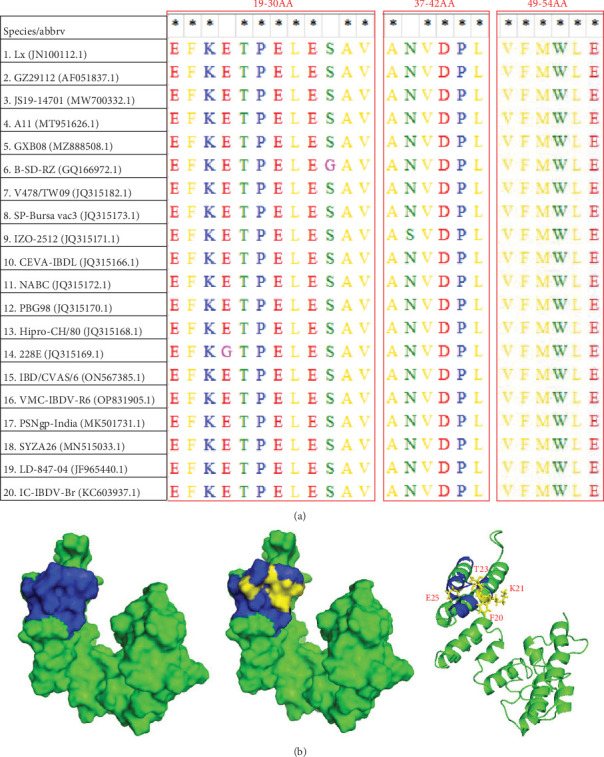
Bioinformatics analysis of the novel antigenic epitopes of VP3 protein. (A) Analysis of conserved epitopes of VP3 gene. The IBDV Lx strain was used as a reference strain, and only residues of the antigenic epitope region are shown. (B) Spatial distribution of the antigenic epitopes recognized by mAb 19D8 on the VP3 protein. The model template was selected from the Protein Data Bank (PDB) library. Blue: key antigenic epitopes recognized by mAb 19D8. Yellow: Essential amino acids in the antigenic epitope region.

Next, the VP3 protein sequences of the 20 different IBDV strains from different construes were analyzed to determine the degree of homology between the IBDV strains (Table 6) and the antigenic epitopes recognized by the mAb 19D8. The results indicated that the four antigenic epitopes were relatively conserved across strains (Figure 5A). The three-dimensional structure of IBDV VP3 was predicted using I-TASSER software, and the antigenic epitopes were recognized by mAb 19D8 in PyMOL. The results demonstrated that all four antigenic epitopes recognized by the antibody were situated on the protein surface and comprised an α-helix structure (Figure 5B). The mAb 19D8 may exert its neutralizing effect via this specific structural feature.

## 4. Discussion

Infectious Bursal disease (IBD) is an acute, virulent, and highly contagious disease of young chickens caused by IBDV infection [24]. Severe immunosuppression occurs in the surviving chickens, which leads to mixed and secondary infections and increased mortality, and causes huge economic losses to the poultry industry [2]. It is currently believed that the IBDV genome encodes five viral proteins. Conventionally, VP2 has been regarded as the primary target for neutralizing antibodies because of its role in host cell receptor binding and its exposure on the viral surface [25]. The VP3 protein, a major structural component of IBDV, plays a critical role in viral assembly and immune evasion [26]. However, our findings demonstrate that VP3, previously considered a structural scaffolding protein with limited immunogenic relevance [20], also harbors functional neutralizing antigenic epitopes. However, this neutralizing effect is strictly confined to the specific strains under investigation. The potential neutralizing efficacy against other strains or genotypes remains to be substantiated through further empirical validation. The discovery of neutralizing antigenic epitopes on the VP3 protein of IBDV represents a significant advancement in our understanding of the immunogenicity of this pathogen, opening new avenues for vaccine development and providing critical insights into the synergistic potential of multiantigenic epitope vaccine strategies.

The identification of VP3-specific mAbs with neutralizing activity suggests that VP3 plays an underappreciated role in IBDV-host interactions. Although VP2 contains hypervariable regions responsible for antigenic drift and immune evasion, VP3's conserved structural role in capsid assembly may render its antigenic epitopes less prone to mutational escape [27]. The key amino acid sites recognized by mAb 19D8 are relatively conserved across various strains. Thus, mAb 19D8 may demonstrate the ability to neutralize a broad spectrum of IBDV strains, in contrast to VP2-directed antibodies, which often exhibit strain-specific limitations.

Much work has been conducted on antigenic epitope studies of IBDV VP2. However, the antigenic epitope of VP3 has not been precisely characterized. A rapid immune response to the linear VP3 antigenic epitope was observed after both immunization and infection, suggesting that VP3 is one of the major immunogens of IBDV [28]. Some studies have reported that the 109–119AA and 177–190AA were located as the antigenic epitopes of VP3 [22]. Two of these antigenic domains (890–910 AA and C-terminal 944 AA) also carry antigenic epitopes that are specific to one serotype; the cross-reacting antigenic epitope is located at 764–885 AA [29]. In this study, we identified a mAb capable of effectively neutralizing viral infection. To accurately locate the antigenic epitopes of the VP3 protein, we expressed the purified recombinant VP3 protein in *E coli* BL21 (DE3), and eight mAbs against the VP3 protein were obtained. These mAbs demonstrated specific reactivity with the VP3 protein of IBDV. Subsequently, using alanine-scanning mutagenesis, the antigenic epitope recognized by 19D8 was mapped to four critical residues (F20, K21, T23, and E25) within the VP3 protein. The identification of VP3 neutralizing antigenic epitopes provides a foundation for developing next-generation vaccines that can co-opt both the VP2 and VP3 antigens. Notably, the inclusion of VP3 may also enhance CD^4+^ T-cell responses because of its conserved T-helper epitopes, which could improve the durability of vaccine-induced immunity [30–32]. However, ensuring the proper folding of tandem antigenic epitopes and avoiding unintended interactions that might mask critical antigenic sites remain challenging [33–35].

A key limitation of VP2-focused vaccines is spatial competition between antibodies targeting adjacent antigenic epitopes of VP2 [36]. The hypervariable regions of VP2 contain multiple overlapping epitopes, and steric hindrance between antibodies binding to these regions can reduce neutralizing efficiency [37, 38]. For example, conformational epitopes in the VP2 puff domain may mask or destabilize neighboring epitopes, thus limiting the simultaneous engagement of multiple antibodies [39]. In contrast, VP3 antigenic epitopes are spatially distinct from VP2 This spatial separation suggests that VP2 and VP3 antigenic epitopes could function additively or synergistically without steric interference [36, 40]. The discovery of VP3 neutralizing antigenic epitopes raises intriguing questions regarding the evolution of IBDV. For instance, if VP3 is under immune pressure in natural infections, why does it remain relatively conserved compared to VP2? One hypothesis that can answer this question is that VP3's essential role in capsid assembly imposes strong functional constraints, limiting its mutation capacity [41]. Alternatively, the immune system may prioritize VP2 due to its surface exposure, allowing VP3 antigenic epitopes to remain “cryptic” and less subject to selective pressure. Surveillance of field strains for VP3 mutations following general use of VP3-containing vaccines will be essential to detect possible escape variants. Although our findings highlight the potential of VP3 as a vaccine candidate, further studies are needed for the structural characterization of VP3 and in vivo validation of VP2-VP3 multiantigenic epitope vaccines.

In conclusion, this study reveals the potential of VP3 as a novel neutralizing antigenic epitope and suggests future research directions for VP2–VP3 multiantigenic epitope vaccines. By incorporating VP3 into multiantigenic epitope vaccine platforms, we can overcome the limitations of current VP2-centric strategies and develop more robust and sustainable solutions against this economically significant avian pathogen. Although steric hindrance and other problems still need to be overcome, through the cross-innovation of structural biology and immunology, multiantigenic epitope vaccines are expected to become a breakthrough tool for dealing with viral variation and immune escape. Future research needs to find a balance between in-depth mechanistic analysis and technological transformation to ultimately achieve the clinical application of broad-spectrum and efficient vaccines.

## Figures and Tables

**Table 1:** Primers used in this study.

Plasmids	Primers (5′-3′)
His-VP3	F: ATCGGATCCGAATTCATGCCATACCTTCCA
	R: GTGGTGGTGCTCGAGATGCTTCATCTCCAT

**Table 2:** Homologous recombination system.

Reagent	Volume
Vector	108 ng
5 × CE II buffer	4 μL
Desired gene	11.52 ng
Exnase II	2 μL
ddH_2_O	Add to 20 μL

**Table 3:** Primers used in the RT-qPCR analysis.

Gene name	Primers (5′-3′)
Gullas-*VP3*	F: TATGGAAGCAGCAGCCAATG
	R: TCGGGTCGCTGAGTGCAAAG

**Table 4:** Sequences of primers used to construct mutants.

Plasmids	Primers (5′-3′)	Positions (amino acid)
VP3-1	**F** GCAGCCGCTGCAGCCGCTGCAGGACGCCAGTACCACCTTGCCATG	1–6AA
	**R** AGCGGCTGCAGCGGCTGCCATGGTCGACCCCTTGGCATCGTCGTC	
VP3-2	**F** GCAGCAGCCGCGGCCGCCCTTGCCATGGCTGCATCAGAGTTCAAAG	7–12AA
	**R** GGCGGCCGCGGCTGCTGCATTGGGTGGAAGGTATGGCATGGTCGAC	
VP3-3	**F** GCTGCCGCGGCTGCAGCAGAGTTCAAAGAGACCCCCGAACTCGAG	13–18AA
	**R** TGCTGCAGCCGCGGCAGCGTGGTACTGGCGTCCTGCATTGGGTGG	
VP3-4	**F** GCGGCCGCAGCGGCCGCCGAACTCGAGAGTGCCGTCAGAGCAATG	19–24AA
	**R** GGCGGCCGCTGCGGCCGCTGATGCAGCCATGGCAAGGTGGTACTG	
VP3-5	**F** GCAGCCGCGGCTGCCGCCAGAGCAATGGAAGCAGCAGCCAACGTG	25–30AA
	**R** GGCGGCAGCCGCGGCTGCGGGGGTCTCTTTGAACTCTGATGCAGC	
VP3-6	**F** GCAGCAGCGGCAGCAGCAGCCAACGTGGACCCACTATTCCAATCTG	31–36AA
	**R** TGCTGCTGCCGCTGCTGCGACGGCACTCTCGAGTTCGGGGGTCTC	
VP3-7	**F** GCCGCCGCGGCCGCAGCATTCCAATCTGCACTCAGTGTGTTCATG	37–42AA
	**R** TGCTGCGGCCGCGGCGGCTGCTGCTTCCATTGCTCTGACGGCACTC	
VP3-8	**F** GCCGCAGCTGCAGCCGCTGTGTTCATGTGGCTGGAAGAGAATGGG	43–48AA
	**R** AGCGGCTGCAGCTGCGGCTAGTGGGTCCACGTTGGCTGCTGCTTC	
VP3-9	**F** GCGGCCGCGGCGGCGGCAGAGAATGGGATTGTGACTGACATGGCC	49–54AA
	**R** TGCCGCCGCCGCGGCCGCACTGAGTGCAGATTGGAATAGTGGGTC	
VP3-10	**F** GCGGCTGCGGCTGCGGCTGACATGGCCAACTTCGCACTCAGCGAC	55–60AA
	**R** AGCCGCAGCCGCAGCCGCTTCCAGCCACATGAACACACTGAGTGC	
VP3-11	**F** GCCGCGGCCGCCGCCGCACTCAGCGACCCGAACGCCCATCGGATG	61–66AA
	**R** TGCGGCGGCGGCCGCGGCAGTCACAATCCCATTCTCTTCCAGCCAC	
VP3-12	**F** GCCGCCGCCGCGGCCGCCCATCGGATGCGAAATTTTCTTGCAAAC	67–72AA
	**R** GGCGGCCGCGGCGGCGGCTGCGAAGTTGGCCATGTCAGTCACAATC	
VP3-13	**F** GCTGCGGCGGCAGCTGCTCTTGCAAACGCACCACAAGCAGGCAGC	73–78AA
	**R** AGCAGCTGCCGCCGCAGCGGCGTTCGGGTCGCTGAGTGCGAAGTTG	
VP3-14	**F** GCTGCAGCCGCAGCAGCAGCAGGCAGCAAGTCGCAAAGGGCCAAG	79–84AA
	**R** TGCTGCTGCGGCTGCAGCAAAATTTCGCATCCGATGGGCGTTCGG	
VP3-15	**F** GCAGCCGCCGCGGCGGCAAGGGCCAAGTACGGGACAGCAGGCTAC	85–90AA
	**R** TGCCGCCGCGGCGGCTGCTTGTGGTGCGTTTGCAAGAAAATTTCG	
VP3-16	**F** GCGGCCGCGGCCGCGGCAGCAGGCTACGGAGTGGAGGCTCGGGGC	91–96AA
	**R** TGCCGCGGCCGCGGCCGCTTGCGACTTGCTGCCTGCTTGTGGTGC	
VP3-17	**F** GCAGCCGCCGGAGCGGCGGCTCGGGGCCCCACACCAGAGGAAGCAC	97–102AA
	**R** CGCCGCTGCGGCGGCTGCTGTCCCGTACTTGGCCCTTTGCGACTTG	
VP3-18	**F** GCTGCGGCCGCCGCAGCAGAGGAAGCACAGAGGGAAAAAGACACAC	103–108AA
	**R** TGCTGCGGCGGCCGCAGCCTCCACTCCGTAGCCTGCTGTCCCGTAC	
VP3-19	**F** GCGGCAGCAGCGGCGGCAAAAGACACACGGATCTCAAAGAAGATG	109–114AA
	**R** TGCCGCCGCTGCTGCCGCTGGTGTGGGGCCCCGAGCCTCCACTCC	
VP3-20	**F** GCAGCCGCAGCGGCCGCAAAGAAGATGGAGACCATGGGCATCTAC	115–120AA
	**R** TGCGGCCGCTGCGGCTGCTTCCCTCTGTGCTTCCTCTGGTGTGGG	
VP3-21	**F** GCGGCGGCGGCGGCCGCGGGCATCTACTTTGCAACACCAGAATGG	121–126AA
	**R** CGCGGCCGCCGCCGCCGCTGAGATCCGTGTGTCTTTTTCCCTCTG	
VP3-22	**F** GCCGCCGCCGCTGCAGCACCAGAATGGGTAGCACTCAATGGGCAC	127–132AA
	**R** TGCTGCAGCGGCGGCGGCCATGGTCTCCATCTTCTTTGAGATCCG	
VP3-23	**F** GCAGCAGCGGCAGCAGCCAATGGGCACCGAGGGCCAAGCCCCGGC	133–138AA
	**R** GGCTGCTGCCGCTGCTGCTGTTGCAAAGTAGATGCCCATGGTCTC	
VP3-24	**F** GCTGCGGCCGCAGCGGCAAGCCCCGGCCAGCTAAAGTACTGGCAG	139–144AA
	**R** TGCCGCTGCGGCCGCAGCGAGTGCTACCCATTCTGGTGTTGCAAAG	
VP3-25	**F** GCCGCCGCCGCGGCAGCGTACTGGCAGAACACACGAGAAATACCG	145–150AA
	**R** CGCTGCCGCGGCGGCGGCTGGCCCTCGGTGCCCATTGAGTGCTAC	
VP3-26	**F** GCCGCGGCGGCCGCAGCAGAAATACCGGACCCAAACGAGGACTATC	151–156AA
	**R** TGCTGCGGCCGCCGCGGCCTTTAGCTGGCCGGGGCTTGGCCCTCG	
VP3-27	**F** GCAGCAGCGGCCGCAGCCGAGGACTATCTAGACTACGTGCATGCAG	157–162AA
	**R** GGCTGCGGCCGCTGCTGCTCGTGTGTTCTGCCAGTACTTTAGCTG	
VP3-28	**F** GCGGCCGCTGCAGCCGCCGTGCATGCAGAGAAGAGCCGGTTGGC	163–168AA
	**R** GGCGGCTGCAGCGGCCGCGTTTGGGTCCGGTATTTCTCGTGTGTTC	
VP3-29	**F** GCGGCTGCAGCGGCGGCCCGGTTGGCATCAGAAGAACAAATCCTAAG	169–174AA
	**R** GGCCGCCGCTGCAGCCGCGTAGTCTAGATAGTCCTCGTTTGGGTC	
VP3-30	**F** GCGGCGGCAGCAGCAGCACAAATCCTAAGGGCAGCTACGTCGATC	175–180AA
	**R** TGCTGCTGCTGCCGCCGCGCTCTTCTCTGCATGCACGTAGTCTAG	
VP3-31	**F** GCAGCCGCAGCGGCAGCTACGTCGATCTACGGGGCTCCAGGACAG	181–186AA
	**R** AGCTGCCGCTGCGGCTGCTTCTTCTGATGCCAACCGGCTCTTCTC	
VP3-32	**F** GCGGCGGCCGCCGCGGCTCCAGGACAGGCAGAGCCACCCCAAGC	187–192AA
	**R** AGCCGCGGCGGCCGCCGCAGCTGCCCTTAGGATTTGTTCTTCTG	
VP3-33	**F** GCAGCAGCGGCAGCGGCACCCCAAGCTTTCATAGACGAAGTTGCC	193–198AA
	**R** TGCCGCTGCCGCTGCTGCAGCCCCGTAGATCGACGTAGCTGCCC	
VP3-34	**F** GCCGCAGCTGCCGCAGCCGAAGTTGCCAAAGTCTATGAAATCAAC	199–204AA
	**R** GGCTGCGGCAGCTGCGGCTGGCTCTGCCTGTCCTGGAGCCCCGTAG	
VP3-35	**F** GCAGCTGCCGCAGCCGCTGAAATCAACCATGGACGTGGCCCAAAC	205–210AA
	**R** AGCGGCTGCGGCAGCTGCGTCTATGAAAGCTTGGGGTGGCTCTGC	
VP3-36	**F** GCAGCCGCCGCTGGAGCTGGCCCAAACCAAGAACAGATGAAAGATC	211–216AA
	**R** AGCTGCAGCGGCGGCTGCATAGACTTTGGCAACTTCGTCTATGAAAG	
VP3-37	**F** GCCGCAGCCGCAGCAGCGATGAAAGATCTGCTCTTGACTGCGATG	217–222AA
	**R** CGCTGCTGCGGCTGCGGCACGTCCATGGTTGATTTCATAGACTTTG	
VP3-38	**F** GCGGCAGCTGCGGCCGCGACTGCGATGGAGATGAAGCATGCGGCC	223–228AA
	**R** CGCGGCCGCAGCTGCCGCCTGTTCTTGGTTTGGGCCACGTCCATG	
VP3-39	**F** GCTGCGGCGGCGGCGGCGCATGCGGCCGCTCGAGCATGCATCTAG	229–235AA
	**R** AGCCGCCGCCGCCGCCGCAGTCAAGAGCAGATCTTTCATCTGTTC	
VP3-4-1	**F** TCAGCGTTCAAAGAGACCCCCG	19AA
	**R** GAACGCTGATGCAGCCATGGCAAG	
VP3-4-2	**F** GAGGCCAAAGAGACCCCCGAACTC	20AA
	**R** TTTGGCCTCTGATGCAGCCATG	
VP3-4-3	**F** TTCGCAGAGACCCCCGAACTCGAG	21AA
	**R** CTCTGCGAACTCTGATGCAGCC	
VP3-4-4	**F** AAAGCGACCCCCGAACTCGAGAG	22AA
	**R** GGTCGCTTTGAACTCTGATGCAG	
VP3-4-5	**F** GAGGCCCCCGAACTCGAGAGTGCC	23AA
	**R** GGGGGCCTCTTTGAACTCTGATGC	
VP3-4-6	**F** ACCGCCGAACTCGAGAGTGCCGTC	24AA
	**R** TTCGGCGGTCTCTTTGAACTCTGATG	
VP3-5-1	**F** CCCGCACTCGAGAGTGCCGTCAGAG	25AA
	**R** GAGTGCGGGGGTCTCTTTGAACTCTG	
VP3-5-2	**F** GAAGCCGAGAGTGCCGTCAGAGCAATG	26AA
	**R** CTCGGCTTCGGGGGTCTCTTTGAAC	
VP3-5-3	**F** CTCGCGAGTGCCGTCAGAGCAATGG	27AA
	**R** ACTCGCGAGTTCGGGGGTCTCTTTG	
VP3-5-4	**F** GAGGCTGCCGTCAGAGCAATGGAAG	28AA
	**R** GGCAGCCTCGAGTTCGGGGGTCTC	
VP3-5-5	**F** AGTGTCGTCAGAGCAATGGAAGCAG	29AA
	**R** GACGACACTCTCGAGTTCGGGGGTC	
VP3-5-6	**F** GCCGCCAGAGCAATGGAAGCAGCAG	30AA
	**R** TCTGGCGGCACTCTCGAGTTCGGGG	
VP3-7-1	**F** GCAGCAGGCAACGTGGACCCACTATTCCAATCTGCAC	37AA
	**R** CACGTTGGGTGCTGCTTCCATTGCTCTGACGGCACTC	
VP3-7-2	**F** GCAGCCGCCGTGGACCCACTATTCCAATCTGCACTC	38AA
	**R** GTCCACGGCGGCTGCTGCTTCCATTGCTCTGACGGCAC	
VP3-7-3	**F** GCCAACGCGGACCCACTATTCCAATCTGCACTCAGTG	39AA
	**R** TGGGTCCGCGTTGGCTGCTGCTTCCATTGCTCTGAC	
VP3-7-4	**F** AACGTGGCCCCACTATTCCAATCTGCACTCAGTGTG	40AA
	**R** TAGTGGGGCCACGTTGGCTGCTGCTTCCATTGCTCTG	
VP3-7-5	**F** GTGGACGCACTATTCCAATCTGCACTCAGTGTGTTC	41AA
	**R** GAATAGTGCGTCCACGTTGGCTGCTGCTTCCATTGC	
VP3-7-6	**F** GACCCAGCATTCCAATCTGCACTCAGTGTGTTCATG	42AA
	**R** TTGGAATGCTGGGTCCACGTTGGCTGCTGCTTCCATTG	
VP3-9-1	**F** CTCAGTGCGTTCATGTGGCTGGAAGAGAATGGGATTG	49AA
	**R** CATGAACGCACTGAGTGCAGATTGGAATAGTGGGTC	
VP3-9-2	**F** AGTGTGGCCATGTGGCTGGAAGAGAATGGGATTGTG	50AA
	**R** CCACATGGCCACACTGAGTGCAGATTGGAATAGTGG	
VP3-9-3	**F** GTGTTCGCGTGGCTGGAAGAGAATGGGATTGTGACTG	51AA
	**R** CAGCCACGCGAACACACTGAGTGCAGATTGGAATAG	
VP3-9-4	**F** TTCATGGCGCTGGAAGAGAATGGGATTGTGACTGAC	52AA
	**R** TTCCAGCGCCATGAACACACTGAGTGCAGATTGGAATAG	
VP3-9-5	**F** ATGTGGGCGGAAGAGAATGGGATTGTGACTGACATG	53AA
	**R** CTCTTCCGCCCACATGAACACACTGAGTGCAGATTG	
VP3-9-6	**F** TGGCTGGCAGAGAATGGGATTGTGACTGACATGGCC	54AA
	**R** ATTCTCTGCCAGCCACATGAACACACTGAGTGCAGATTG	

**Table 5:** Amino acid sequence of the VP3 mutant.

Title	Amino Acid Sequence	Positions (amino acid)
VP3	PYLPPNAGRQYHLAMAASEFKETPELESAVRAMEAAANVDPLFQSALSVFMWLEENGIVTDMANFALSDPNAHRMRNFLANAPQAGSKSQRAKYGTAGYGVEARGPTPEEAQREKDTRISKKMETMGIYFATPEWVALNGHRGPSPGQLKYWQNTREIPDPNEDYLDYVHAEKSRLASEEQILRAATSIYGAPGQAEPPQAFIDEVAKVYEINHGRGPNQEQMKDLLLTAMEMKH	1–235AA

VP3-1	AAAAAAAGRQYHLAMAASEFKETPELESAVRAMEAAANVDPLFQSALSVFMWLEENGIVTDMANFALSDPNAHRMRNFLANAPQAGSKSQRAKYGTAGYGVEARGPTPEEAQREKDTRISKKMETMGIYFATPEWVALNGHRGPSPGQLKYWQNTREIPDPNEDYLDYVHAEKSRLASEEQILRAATSIYGAPGQAEPPQAFIDEVAKVYEINHGRGPNQEQMKDLLLTAMEMKH	1–6AA

VP3-2	PYLPPNGAAAAALAMAASEFKETPELESAVRAMEAAANVDPLFQSALSVFMWLEENGIVTDMANFALSDPNAHRMRNFLANAPQAGSKSQRAKYGTAGYGVEARGPTPEEAQREKDTRISKKMETMGIYFATPEWVALNGHRGPSPGQLKYWQNTREIPDPNEDYLDYVHAEKSRLASEEQILRAATSIYGAPGQAEPPQAFIDEVAKVYEINHGRGPNQEQMKDLLLTAMEMKH	7–12AA

VP3-3	PYLPPNAGRQYHAGAGGAEFKETPELESAVRAMEAAANVDPLFQSALSVFMWLEENGIVTDMANFALSDPNAHRMRNFLANAPQAGSKSQRAKYGTAGYGVEARGPTPEEAQREKDTRISKKMETMGIYFATPEWVALNGHRGPSPGQLKYWQNTREIPDPNEDYLDYVHAEKSRLASEEQILRAATSIYGAPGQAEPPQAFIDEVAKVYEINHGRGPNQEQMKDLLLTAMEMKH	13–18AA

VP3-4	PYLPPNAGRQYHLAMAASAAAAAAELESAVRAMEAAANVDPLFQSALSVFMWLEENGIVTDMANFALSDPNAHRMRNFLANAPQAGSKSQRAKYGTAGYGVEARGPTPEEAQREKDTRISKKMETMGIYFATPEWVALNGHRGPSPGQLKYWQNTREIPDPNEDYLDYVHAEKSRLASEEQILRAATSIYGAPGQAEPPQAFIDEVAKVYEINHGRGPNQEQMKDLLLTAMEMKH	19–24AA

VP3-5	PYLPPNAGRQYHLAMAASEFKETPAAAAGARAMEAAANVDPLFQSALSVFMWLEENGIVTDMANFALSDPNAHRMRNFLANAPQAGSKSQRAKYGTAGYGVEARGPTPEEAQREKDTRISKKMETMGIYFATPEWVALNGHRGPSPGQLKYWQNTREIPDPNEDYLDYVHAEKSRLASEEQILRAATSIYGAPGQAEPPQAFIDEVAKVYEINHGRGPNQEQMKDLLLTAMEMKH	25–30AA

VP3-6	PYLPPNAGRQYHLAMAASEFKETPELESAVAGAAGGANVDPLFQSALSVFMWLEENGIVTDMANFALSDPNAHRMRNFLANAPQAGSKSQRAKYGTAGYGVEARGPTPEEAQREKDTRISKKMETMGIYFATPEWVALNGHRGPSPGQLKYWQNTREIPDPNEDYLDYVHAEKSRLASEEQILRAATSIYGAPGQAEPPQAFIDEVAKVYEINHGRGPNQEQMKDLLLTAMEMKH	31–36AA

VP3-7	PYLPPNAGRQYHLAMAASEFKETPELESAVRAMEAAGAAAAAFQSALSVFMWLEENGIVTDMANFALSDPNAHRMRNFLANAPQAGSKSQRAKYGTAGYGVEARGPTPEEAQREKDTRISKKMETMGIYFATPEWVALNGHRGPSPGQLKYWQNTREIPDPNEDYLDYVHAEKSRLASEEQILRAATSIYGAPGQAEPPQAFIDEVAKVYEINHGRGPNQEQMKDLLLTAMEMKH	37–42AA

VP3-8	PYLPPNAGRQYHLAMAASEFKETPELESAVRAMEAAANVDPLAAAGAAVFMWLEENGIVTDMANFALSDPNAHRMRNFLANAPQAGSKSQRAKYGTAGYGVEARGPTPEEAQREKDTRISKKMETMGIYFATPEWVALNGHRGPSPGQLKYWQNTREIPDPNEDYLDYVHAEKSRLASEEQILRAATSIYGAPGQAEPPQAFIDEVAKVYEINHGRGPNQEQMKDLLLTAMEMKH	43–48AA

VP3-9	PYLPPNAGRQYHLAMAASEFKETPELESAVRAMEAAANVDPLFQSALSAAAAAAENGIVTDMANFALSDPNAHRMRNFLANAPQAGSKSQRAKYGTAGYGVEARGPTPEEAQREKDTRISKKMETMGIYFATPEWVALNGHRGPSPGQLKYWQNTREIPDPNEDYLDYVHAEKSRLASEEQILRAATSIYGAPGQAEPPQAFIDEVAKVYEINHGRGPNQEQMKDLLLTAMEMKH	49–54AA

VP3-10	PYLPPNAGRQYHLAMAASEFKETPELESAVRAMEAAANVDPLFQSALSVFMWLEAAAAAADMANFALSDPNAHRMRNFLANAPQAGSKSQRAKYGTAGYGVEARGPTPEEAQREKDTRISKKMETMGIYFATPEWVALNGHRGPSPGQLKYWQNTREIPDPNEDYLDYVHAEKSRLASEEQILRAATSIYGAPGQAEPPQAFIDEVAKVYEINHGRGPNQEQMKDLLLTAMEMKH	55–60AA

VP3-11	PYLPPNAGRQYHLAMAASEFKETPELESAVRAMEAAANVDPLFQSALSVFMWLEENGIVTAAGAAGLSDPNAHRMRNFLANAPQAGSKSQRAKYGTAGYGVEARGPTPEEAQREKDTRISKKMETMGIYFATPEWVALNGHRGPSPGQLKYWQNTREIPDPNEDYLDYVHAEKSRLASEEQILRAATSIYGAPGQAEPPQAFIDEVAKVYEINHGRGPNQEQMKDLLLTAMEMKH	61–66AA

VP3-12	PYLPPNAGRQYHLAMAASEFKETPELESAVRAMEAAANVDPLFQSALSVFMWLEENGIVTDMANFAAAAAAGHRMRNFLANAPQAGSKSQRAKYGTAGYGVEARGPTPEEAQREKDTRISKKMETMGIYFATPEWVALNGHRGPSPGQLKYWQNTREIPDPNEDYLDYVHAEKSRLASEEQILRAATSIYGAPGQAEPPQAFIDEVAKVYEINHGRGPNQEQMKDLLLTAMEMKH	67–72AA

VP3-13	PYLPPNAGRQYHLAMAASEFKETPELESAVRAMEAAANVDPLFQSALSVFMWLEENGIVTDMANFALSDPNAAAAAAALANAPQAGSKSQRAKYGTAGYGVEARGPTPEEAQREKDTRISKKMETMGIYFATPEWVALNGHRGPSPGQLKYWQNTREIPDPNEDYLDYVHAEKSRLASEEQILRAATSIYGAPGQAEPPQAFIDEVAKVYEINHGRGPNQEQMKDLLLTAMEMKH	73–78AA

VP3-14	PYLPPNAGRQYHLAMAASEFKETPELESAVRAMEAAANVDPLFQSALSVFMWLEENGIVTDMANFALSDPNAHRMRNFAGAGAAAGSKSQRAKYGTAGYGVEARGPTPEEAQREKDTRISKKMETMGIYFATPEWVALNGHRGPSPGQLKYWQNTREIPDPNEDYLDYVHAEKSRLASEEQILRAATSIYGAPGQAEPPQAFIDEVAKVYEINHGRGPNQEQMKDLLLTAMEMKH	79–84AA

VP3-15	PYLPPNAGRQYHLAMAASEFKETPELESAVRAMEAAANVDPLFQSALSVFMWLEENGIVTDMANFALSDPNAHRMRNFLANAPQGAAAAARAKYGTAGYGVEARGPTPEEAQREKDTRISKKMETMGIYFATPEWVALNGHRGPSPGQLKYWQNTREIPDPNEDYLDYVHAEKSRLASEEQILRAATSIYGAPGQAEPPQAFIDEVAKVYEINHGRGPNQEQMKDLLLTAMEMKH	85–90AA

VP3-16	PYLPPNAGRQYHLAMAASEFKETPELESAVRAMEAAANVDPLFQSALSVFMWLEENGIVTDMANFALSDPNAHRMRNFLANAPQAGSKSQAGAAAAAGYGVEARGPTPEEAQREKDTRISKKMETMGIYFATPEWVALNGHRGPSPGQLKYWQNTREIPDPNEDYLDYVHAEKSRLASEEQILRAATSIYGAPGQAEPPQAFIDEVAKVYEINHGRGPNQEQMKDLLLTAMEMKH	91–96AA

VP3-17	PYLPPNAGRQYHLAMAASEFKETPELESAVRAMEAAANVDPLFQSALSVFMWLEENGIVTDMANFALSDPNAHRMRNFLANAPQAGSKSQRAKYGTGAAAAAARGPTPEEAQREKDTRISKKMETMGIYFATPEWVALNGHRGPSPGQLKYWQNTREIPDPNEDYLDYVHAEKSRLASEEQILRAATSIYGAPGQAEPPQAFIDEVAKVYEINHGRGPNQEQMKDLLLTAMEMKH	97–102AA

VP3-18	PYLPPNAGRQYHLAMAASEFKETPELESAVRAMEAAANVDPLFQSALSVFMWLEENGIVTDMANFALSDPNAHRMRNFLANAPQAGSKSQRAKYGTAGYGVEGAAAAAEEAQREKDTRISKKMETMGIYFATPEWVALNGHRGPSPGQLKYWQNTREIPDPNEDYLDYVHAEKSRLASEEQILRAATSIYGAPGQAEPPQAFIDEVAKVYEINHGRGPNQEQMKDLLLTAMEMKH	103–108AA

VP3-19	PYLPPNAGRQYHLAMAASEFKETPELESAVRAMEAAANVDPLFQSALSVFMWLEENGIVTDMANFALSDPNAHRMRNFLANAPQAGSKSQRAKYGTAGYGVEARGPTPAAGAAAKDTRISKKMETMGIYFATPEWVALNGHRGPSPGQLKYWQNTREIPDPNEDYLDYVHAEKSRLASEEQILRAATSIYGAPGQAEPPQAFIDEVAKVYEINHGRGPNQEQMKDLLLTAMEMKH	109–114AA

VP3-20	PYLPPNAGRQYHLAMAASEFKETPELESAVRAMEAAANVDPLFQSALSVFMWLEENGIVTDMANFALSDPNAHRMRNFLANAPQAGSKSQRAKYGTAGYGVEARGPTPEEAQREAAAAAAKKMETMGIYFATPEWVALNGHRGPSPGQLKYWQNTREIPDPNEDYLDYVHAEKSRLASEEQILRAATSIYGAPGQAEPPQAFIDEVAKVYEINHGRGPNQEQMKDLLLTAMEMKH	115–120AA

VP3-21	PYLPPNAGRQYHLAMAASEFKETPELESAVRAMEAAANVDPLFQSALSVFMWLEENGIVTDMANFALSDPNAHRMRNFLANAPQAGSKSQRAKYGTAGYGVEARGPTPEEAQREKDTRISAAAAAAGIYFATPEWVALNGHRGPSPGQLKYWQNTREIPDPNEDYLDYVHAEKSRLASEEQILRAATSIYGAPGQAEPPQAFIDEVAKVYEINHGRGPNQEQMKDLLLTAMEMKH	121–126AA

VP3-22	PYLPPNAGRQYHLAMAASEFKETPELESAVRAMEAAANVDPLFQSALSVFMWLEENGIVTDMANFALSDPNAHRMRNFLANAPQAGSKSQRAKYGTAGYGVEARGPTPEEAQREKDTRISKKMETMAAAAGAPEWVALNGHRGPSPGQLKYWQNTREIPDPNEDYLDYVHAEKSRLASEEQILRAATSIYGAPGQAEPPQAFIDEVAKVYEINHGRGPNQEQMKDLLLTAMEMKH	127–132AA

VP3-23	PYLPPNAGRQYHLAMAASEFKETPELESAVRAMEAAANVDPLFQSALSVFMWLEENGIVTDMANFALSDPNAHRMRNFLANAPQAGSKSQRAKYGTAGYGVEARGPTPEEAQREKDTRISKKMETMGIYFATAAAAGANGHRGPSPGQLKYWQNTREIPDPNEDYLDYVHAEKSRLASEEQILRAATSIYGAPGQAEPPQAFIDEVAKVYEINHGRGPNQEQMKDLLLTAMEMKH	133–138AA

VP3-24	PYLPPNAGRQYHLAMAASEFKETPELESAVRAMEAAANVDPLFQSALSVFMWLEENGIVTDMANFALSDPNAHRMRNFLANAPQAGSKSQRAKYGTAGYGVEARGPTPEEAQREKDTRISKKMETMGIYFATPEWVALAAAAAASPGQLKYWQNTREIPDPNEDYLDYVHAEKSRLASEEQILRAATSIYGAPGQAEPPQAFIDEVAKVYEINHGRGPNQEQMKDLLLTAMEMKH	139–144AA

VP3-25	PYLPPNAGRQYHLAMAASEFKETPELESAVRAMEAAANVDPLFQSALSVFMWLEENGIVTDMANFALSDPNAHRMRNFLANAPQAGSKSQRAKYGTAGYGVEARGPTPEEAQREKDTRISKKMETMGIYFATPEWVALNGHRGPAAAAAAYWQNTREIPDPNEDYLDYVHAEKSRLASEEQILRAATSIYGAPGQAEPPQAFIDEVAKVYEINHGRGPNQEQMKDLLLTAMEMKH	145–150AA

VP3-26	PYLPPNAGRQYHLAMAASEFKETPELESAVRAMEAAANVDPLFQSALSVFMWLEENGIVTDMANFALSDPNAHRMRNFLANAPQAGSKSQRAKYGTAGYGVEARGPTPEEAQREKDTRISKKMETMGIYFATPEWVALNGHRGPSPGQLKAAAAAAEIPDPNEDYLDYVHAEKSRLASEEQILRAATSIYGAPGQAEPPQAFIDEVAKVYEINHGRGPNQEQMKDLLLTAMEMKH	151–156AA

VP3-27	PYLPPNAGRQYHLAMAASEFKETPELESAVRAMEAAANVDPLFQSALSVFMWLEENGIVTDMANFALSDPNAHRMRNFLANAPQAGSKSQRAKYGTAGYGVEARGPTPEEAQREKDTRISKKMETMGIYFATPEWVALNGHRGPSPGQLKYWQNTRAAAAAAEDYLDYVHAEKSRLASEEQILRAATSIYGAPGQAEPPQAFIDEVAKVYEINHGRGPNQEQMKDLLLTAMEMKH	157–162AA

VP3-28	PYLPPNAGRQYHLAMAASEFKETPELESAVRAMEAAANVDPLFQSALSVFMWLEENGIVTDMANFALSDPNAHRMRNFLANAPQAGSKSQRAKYGTAGYGVEARGPTPEEAQREKDTRISKKMETMGIYFATPEWVALNGHRGPSPGQLKYWQNTREIPDPNAAAAAAVHAEKSRLASEEQILRAATSIYGAPGQAEPPQAFIDEVAKVYEINHGRGPNQEQMKDLLLTAMEMKH	163–168AA

VP3-29	PYLPPNAGRQYHLAMAASEFKETPELESAVRAMEAAANVDPLFQSALSVFMWLEENGIVTDMANFALSDPNAHRMRNFLANAPQAGSKSQRAKYGTAGYGVEARGPTPEEAQREKDTRISKKMETMGIYFATPEWVALNGHRGPSPGQLKYWQNTREIPDPNEDYLDYAAGAAARLASEEQILRAATSIYGAPGQAEPPQAFIDEVAKVYEINHGRGPNQEQMKDLLLTAMEMKH	169–174AA

VP3-30	PYLPPNAGRQYHLAMAASEFKETPELESAVRAMEAAANVDPLFQSALSVFMWLEENGIVTDMANFALSDPNAHRMRNFLANAPQAGSKSQRAKYGTAGYGVEARGPTPEEAQREKDTRISKKMETMGIYFATPEWVALNGHRGPSPGQLKYWQNTREIPDPNEDYLDYVHAEKSAAGAAAQILRAATSIYGAPGQAEPPQAFIDEVAKVYEINHGRGPNQEQMKDLLLTAMEMKH	175–180AA

VP3-31	PYLPPNAGRQYHLAMAASEFKETPELESAVRAMEAAANVDPLFQSALSVFMWLEENGIVTDMANFALSDPNAHRMRNFLANAPQAGSKSQRAKYGTAGYGVEARGPTPEEAQREKDTRISKKMETMGIYFATPEWVALNGHRGPSPGQLKYWQNTREIPDPNEDYLDYVHAEKSRLASEEAAAAGGTSIYGAPGQAEPPQAFIDEVAKVYEINHGRGPNQEQMKDLLLTAMEMKH	181–186AA

VP3-32	PYLPPNAGRQYHLAMAASEFKETPELESAVRAMEAAANVDPLFQSALSVFMWLEENGIVTDMANFALSDPNAHRMRNFLANAPQAGSKSQRAKYGTAGYGVEARGPTPEEAQREKDTRISKKMETMGIYFATPEWVALNGHRGPSPGQLKYWQNTREIPDPNEDYLDYVHAEKSRLASEEQILRAAAAAAAGPGQAEPPQAFIDEVAKVYEINHGRGPNQEQMKDLLLTAMEMKH	187–192AA

VP3-33	PYLPPNAGRQYHLAMAASEFKETPELESAVRAMEAAANVDPLFQSALSVFMWLEENGIVTDMANFALSDPNAHRMRNFLANAPQAGSKSQRAKYGTAGYGVEARGPTPEEAQREKDTRISKKMETMGIYFATPEWVALNGHRGPSPGQLKYWQNTREIPDPNEDYLDYVHAEKSRLASEEQILRAATSIYGAAAAGAAPQAFIDEVAKVYEINHGRGPNQEQMKDLLLTAMEMKH	193–198AA

VP3-34	PYLPPNAGRQYHLAMAASEFKETPELESAVRAMEAAANVDPLFQSALSVFMWLEENGIVTDMANFALSDPNAHRMRNFLANAPQAGSKSQRAKYGTAGYGVEARGPTPEEAQREKDTRISKKMETMGIYFATPEWVALNGHRGPSPGQLKYWQNTREIPDPNEDYLDYVHAEKSRLASEEQILRAATSIYGAPGQAEPAAGAAAEVAKVYEINHGRGPNQEQMKDLLLTAMEMKH	199–204AA

VP3-35	PYLPPNAGRQYHLAMAASEFKETPELESAVRAMEAAANVDPLFQSALSVFMWLEENGIVTDMANFALSDPNAHRMRNFLANAPQAGSKSQRAKYGTAGYGVEARGPTPEEAQREKDTRISKKMETMGIYFATPEWVALNGHRGPSPGQLKYWQNTREIPDPNEDYLDYVHAEKSRLASEEQILRAATSIYGAPGQAEPPQAFIDAAGAAAEINHGRGPNQEQMKDLLLTAMEMKH	205–210AA

VP3-36	PYLPPNAGRQYHLAMAASEFKETPELESAVRAMEAAANVDPLFQSALSVFMWLEENGIVTDMANFALSDPNAHRMRNFLANAPQAGSKSQRAKYGTAGYGVEARGPTPEEAQREKDTRISKKMETMGIYFATPEWVALNGHRGPSPGQLKYWQNTREIPDPNEDYLDYVHAEKSRLASEEQILRAATSIYGAPGQAEPPQAFIDEVAKVYAAAAAAGPNQEQMKDLLLTAMEMKH	211–216AA

VP3-37	PYLPPNAGRQYHLAMAASEFKETPELESAVRAMEAAANVDPLFQSALSVFMWLEENGIVTDMANFALSDPNAHRMRNFLANAPQAGSKSQRAKYGTAGYGVEARGPTPEEAQREKDTRISKKMETMGIYFATPEWVALNGHRGPSPGQLKYWQNTREIPDPNEDYLDYVHAEKSRLASEEQILRAATSIYGAPGQAEPPQAFIDEVAKVYEINHGRAAAAAAMKDLLLTAMEMKH	217–222AA

VP3-38	PYLPPNAGRQYHLAMAASEFKETPELESAVRAMEAAANVDPLFQSALSVFMWLEENGIVTDMANFALSDPNAHRMRNFLANAPQAGSKSQRAKYGTAGYGVEARGPTPEEAQREKDTRISKKMETMGIYFATPEWVALNGHRGPSPGQLKYWQNTREIPDPNEDYLDYVHAEKSRLASEEQILRAATSIYGAPGQAEPPQAFIDEVAKVYEINHGRGPNQEQAAAAAATAMEMKH	223–228AA

VP3-39	PYLPPNAGRQYHLAMAASEFKETPELESAVRAMEAAANVDPLFQSALSVFMWLEENGIVTDMANFALSDPNAHRMRNFLANAPQAGSKSQRAKYGTAGYGVEARGPTPEEAQREKDTRISKKMETMGIYFATPEWVALNGHRGPSPGQLKYWQNTREIPDPNEDYLDYVHAEKSRLASEEQILRAATSIYGAPGQAEPPQAFIDEVAKVYEINHGRGPNQEQMKDLLLAGAAAAA	229–235AA

VP3-4-1	PYLPPNAGRQYHLAMAASAFKETPELESAVRAMEAAANVDPLFQSALSVFMWLEENGIVTDMANFALSDPNAHRMRNFLANAPQAGSKSQRAKYGTAGYGVEARGPTPEEAQREKDTRISKKMETMGIYFATPEWVALNGHRGPSPGQLKYWQNTREIPDPNEDYLDYVHAEKSRLASEEQILRAATSIYGAPGQAEPPQAFIDEVAKVYEINHGRGPNQEQMKDLLLTAMEMKH	19AA

VP3-4-2	PYLPPNAGRQYHLAMAASEAKETPELESAVRAMEAAANVDPLFQSALSVFMWLEENGIVTDMANFALSDPNAHRMRNFLANAPQAGSKSQRAKYGTAGYGVEARGPTPEEAQREKDTRISKKMETMGIYFATPEWVALNGHRGPSPGQLKYWQNTREIPDPNEDYLDYVHAEKSRLASEEQILRAATSIYGAPGQAEPPQAFIDEVAKVYEINHGRGPNQEQMKDLLLTAMEMKH	20AA

VP3-4-3	PYLPPNAGRQYHLAMAASEFAETPELESAVRAMEAAANVDPLFQSALSVFMWLEENGIVTDMANFALSDPNAHRMRNFLANAPQAGSKSQRAKYGTAGYGVEARGPTPEEAQREKDTRISKKMETMGIYFATPEWVALNGHRGPSPGQLKYWQNTREIPDPNEDYLDYVHAEKSRLASEEQILRAATSIYGAPGQAEPPQAFIDEVAKVYEINHGRGPNQEQMKDLLLTAMEMKH	21AA

VP3-4-4	PYLPPNAGRQYHLAMAASEFKATPELESAVRAMEAAANVDPLFQSALSVFMWLEENGIVTDMANFALSDPNAHRMRNFLANAPQAGSKSQRAKYGTAGYGVEARGPTPEEAQREKDTRISKKMETMGIYFATPEWVALNGHRGPSPGQLKYWQNTREIPDPNEDYLDYVHAEKSRLASEEQILRAATSIYGAPGQAEPPQAFIDEVAKVYEINHGRGPNQEQMKDLLLTAMEMKH	22AA

VP3-4-5	PYLPPNAGRQYHLAMAASEFKEAPELESAVRAMEAAANVDPLFQSALSVFMWLEENGIVTDMANFALSDPNAHRMRNFLANAPQAGSKSQRAKYGTAGYGVEARGPTPEEAQREKDTRISKKMETMGIYFATPEWVALNGHRGPSPGQLKYWQNTREIPDPNEDYLDYVHAEKSRLASEEQILRAATSIYGAPGQAEPPQAFIDEVAKVYEINHGRGPNQEQMKDLLLTAMEMKH	23AA

VP3-4-6	PYLPPNAGRQYHLAMAASEFKETAELESAVRAMEAAANVDPLFQSALSVFMWLEENGIVTDMANFALSDPNAHRMRNFLANAPQAGSKSQRAKYGTAGYGVEARGPTPEEAQREKDTRISKKMETMGIYFATPEWVALNGHRGPSPGQLKYWQNTREIPDPNEDYLDYVHAEKSRLASEEQILRAATSIYGAPGQAEPPQAFIDEVAKVYEINHGRGPNQEQMKDLLLTAMEMKH	24AA

VP3-5-1	PYLPPNAGRQYHLAMAASEFKETPALESAVRAMEAAANVDPLFQSALSVFMWLEENGIVTDMANFALSDPNAHRMRNFLANAPQAGSKSQRAKYGTAGYGVEARGPTPEEAQREKDTRISKKMETMGIYFATPEWVALNGHRGPSPGQLKYWQNTREIPDPNEDYLDYVHAEKSRLASEEQILRAATSIYGAPGQAEPPQAFIDEVAKVYEINHGRGPNQEQMKDLLLTAMEMKH	25AA

VP3-5-2	PYLPPNAGRQYHLAMAASEFKETPEAESAVRAMEAAANVDPLFQSALSVFMWLEENGIVTDMANFALSDPNAHRMRNFLANAPQAGSKSQRAKYGTAGYGVEARGPTPEEAQREKDTRISKKMETMGIYFATPEWVALNGHRGPSPGQLKYWQNTREIPDPNEDYLDYVHAEKSRLASEEQILRAATSIYGAPGQAEPPQAFIDEVAKVYEINHGRGPNQEQMKDLLLTAMEMKH	26AA

VP3-5-3	PYLPPNAGRQYHLAMAASEFKETPELASAVRAMEAAANVDPLFQSALSVFMWLEENGIVTDMANFALSDPNAHRMRNFLANAPQAGSKSQRAKYGTAGYGVEARGPTPEEAQREKDTRISKKMETMGIYFATPEWVALNGHRGPSPGQLKYWQNTREIPDPNEDYLDYVHAEKSRLASEEQILRAATSIYGAPGQAEPPQAFIDEVAKVYEINHGRGPNQEQMKDLLLTAMEMKH	27AA

VP3-5-4	PYLPPNAGRQYHLAMAASEFKETPELEAAVRAMEAAANVDPLFQSALSVFMWLEENGIVTDMANFALSDPNAHRMRNFLANAPQAGSKSQRAKYGTAGYGVEARGPTPEEAQREKDTRISKKMETMGIYFATPEWVALNGHRGPSPGQLKYWQNTREIPDPNEDYLDYVHAEKSRLASEEQILRAATSIYGAPGQAEPPQAFIDEVAKVYEINHGRGPNQEQMKDLLLTAMEMKH	28AA

VP3-5-5	PYLPPNAGRQYHLAMAASEFKETPELESGVRAMEAAANVDPLFQSALSVFMWLEENGIVTDMANFALSDPNAHRMRNFLANAPQAGSKSQRAKYGTAGYGVEARGPTPEEAQREKDTRISKKMETMGIYFATPEWVALNGHRGPSPGQLKYWQNTREIPDPNEDYLDYVHAEKSRLASEEQILRAATSIYGAPGQAEPPQAFIDEVAKVYEINHGRGPNQEQMKDLLLTAMEMKH	29AA

VP3-5-6	PYLPPNAGRQYHLAMAASEFKETPELESAARAMEAAANVDPLFQSALSVFMWLEENGIVTDMANFALSDPNAHRMRNFLANAPQAGSKSQRAKYGTAGYGVEARGPTPEEAQREKDTRISKKMETMGIYFATPEWVALNGHRGPSPGQLKYWQNTREIPDPNEDYLDYVHAEKSRLASEEQILRAATSIYGAPGQAEPPQAFIDEVAKVYEINHGRGPNQEQMKDLLLTAMEMKH	30AA

VP3-7-1	PYLPPNAGRQYHLAMAASEFKETPELESAVRAMEAAGNVDPLFQSALSVFMWLEENGIVTDMANFALSDPNAHRMRNFLANAPQAGSKSQRAKYGTAGYGVEARGPTPEEAQREKDTRISKKMETMGIYFATPEWVALNGHRGPSPGQLKYWQNTREIPDPNEDYLDYVHAEKSRLASEEQILRAATSIYGAPGQAEPPQAFIDEVAKVYEINHGRGPNQEQMKDLLLTAMEMKH	37AA

VP3-7-2	PYLPPNAGRQYHLAMAASEFKETPELESAVRAMEAAAAVDPLFQSALSVFMWLEENGIVTDMANFALSDPNAHRMRNFLANAPQAGSKSQRAKYGTAGYGVEARGPTPEEAQREKDTRISKKMETMGIYFATPEWVALNGHRGPSPGQLKYWQNTREIPDPNEDYLDYVHAEKSRLASEEQILRAATSIYGAPGQAEPPQAFIDEVAKVYEINHGRGPNQEQMKDLLLTAMEMKH	38AA

VP3-7-3	PYLPPNAGRQYHLAMAASEFKETPELESAVRAMEAAANADPLFQSALSVFMWLEENGIVTDMANFALSDPNAHRMRNFLANAPQAGSKSQRAKYGTAGYGVEARGPTPEEAQREKDTRISKKMETMGIYFATPEWVALNGHRGPSPGQLKYWQNTREIPDPNEDYLDYVHAEKSRLASEEQILRAATSIYGAPGQAEPPQAFIDEVAKVYEINHGRGPNQEQMKDLLLTAMEMKH	39AA

VP3-7-4	PYLPPNAGRQYHLAMAASEFKETPELESAVRAMEAAANVAPLFQSALSVFMWLEENGIVTDMANFALSDPNAHRMRNFLANAPQAGSKSQRAKYGTAGYGVEARGPTPEEAQREKDTRISKKMETMGIYFATPEWVALNGHRGPSPGQLKYWQNTREIPDPNEDYLDYVHAEKSRLASEEQILRAATSIYGAPGQAEPPQAFIDEVAKVYEINHGRGPNQEQMKDLLLTAMEMKH	40AA

VP3-7-5	PYLPPNAGRQYHLAMAASEFKETPELESAVRAMEAAANVDALFQSALSVFMWLEENGIVTDMANFALSDPNAHRMRNFLANAPQAGSKSQRAKYGTAGYGVEARGPTPEEAQREKDTRISKKMETMGIYFATPEWVALNGHRGPSPGQLKYWQNTREIPDPNEDYLDYVHAEKSRLASEEQILRAATSIYGAPGQAEPPQAFIDEVAKVYEINHGRGPNQEQMKDLLLTAMEMKH	41AA

VP3-7-6	PYLPPNAGRQYHLAMAASEFKETPELESAVRAMEAAANVDPAFQSALSVFMWLEENGIVTDMANFALSDPNAHRMRNFLANAPQAGSKSQRAKYGTAGYGVEARGPTPEEAQREKDTRISKKMETMGIYFATPEWVALNGHRGPSPGQLKYWQNTREIPDPNEDYLDYVHAEKSRLASEEQILRAATSIYGAPGQAEPPQAFIDEVAKVYEINHGRGPNQEQMKDLLLTAMEMKH	42AA

VP3-9-1	PYLPPNAGRQYHLAMAASEFKETPELESAVRAMEAAANVDPLFQSALSAFMWLEENGIVTDMANFALSDPNAHRMRNFLANAPQAGSKSQRAKYGTAGYGVEARGPTPEEAQREKDTRISKKMETMGIYFATPEWVALNGHRGPSPGQLKYWQNTREIPDPNEDYLDYVHAEKSRLASEEQILRAATSIYGAPGQAEPPQAFIDEVAKVYEINHGRGPNQEQMKDLLLTAMEMKH	49AA

VP3-9-2	PYLPPNAGRQYHLAMAASEFKETPELESAVRAMEAAANVDPLFQSALSVAMWLEENGIVTDMANFALSDPNAHRMRNFLANAPQAGSKSQRAKYGTAGYGVEARGPTPEEAQREKDTRISKKMETMGIYFATPEWVALNGHRGPSPGQLKYWQNTREIPDPNEDYLDYVHAEKSRLASEEQILRAATSIYGAPGQAEPPQAFIDEVAKVYEINHGRGPNQEQMKDLLLTAMEMKH	50AA

VP3-9-3	PYLPPNAGRQYHLAMAASEFKETPELESAVRAMEAAANVDPLFQSALSVFAWLEENGIVTDMANFALSDPNAHRMRNFLANAPQAGSKSQRAKYGTAGYGVEARGPTPEEAQREKDTRISKKMETMGIYFATPEWVALNGHRGPSPGQLKYWQNTREIPDPNEDYLDYVHAEKSRLASEEQILRAATSIYGAPGQAEPPQAFIDEVAKVYEINHGRGPNQEQMKDLLLTAMEMKH	51AA

VP3-9-4	PYLPPNAGRQYHLAMAASEFKETPELESAVRAMEAAANVDPLFQSALSVFMALEENGIVTDMANFALSDPNAHRMRNFLANAPQAGSKSQRAKYGTAGYGVEARGPTPEEAQREKDTRISKKMETMGIYFATPEWVALNGHRGPSPGQLKYWQNTREIPDPNEDYLDYVHAEKSRLASEEQILRAATSIYGAPGQAEPPQAFIDEVAKVYEINHGRGPNQEQMKDLLLTAMEMKH	52AA

VP3-9-5	PYLPPNAGRQYHLAMAASEFKETPELESAVRAMEAAANVDPLFQSALSVFMWAEENGIVTDMANFALSDPNAHRMRNFLANAPQAGSKSQRAKYGTAGYGVEARGPTPEEAQREKDTRISKKMETMGIYFATPEWVALNGHRGPSPGQLKYWQNTREIPDPNEDYLDYVHAEKSRLASEEQILRAATSIYGAPGQAEPPQAFIDEVAKVYEINHGRGPNQEQMKDLLLTAMEMKH	53AA

VP3-9-6	PYLPPNAGRQYHLAMAASEFKETPELESAVRAMEAAANVDPLFQSALSVFMWLAENGIVTDMANFALSDPNAHRMRNFLANAPQAGSKSQRAKYGTAGYGVEARGPTPEEAQREKDTRISKKMETMGIYFATPEWVALNGHRGPSPGQLKYWQNTREIPDPNEDYLDYVHAEKSRLASEEQILRAATSIYGAPGQAEPPQAFIDEVAKVYEINHGRGPNQEQMKDLLLTAMEMKH	54AA

**Table 6:** IBDV strains included in this study.

Number	Gene accession	Title	Country	Continent	Protein accession
1	JN100112.1	Lx	China	Asia	AEO31527.1
2	AF051837.1	GZ29112	China	Asia	AAC06016.1
3	MW700332.1	IBDV-JS19-14701	China	Asia	UCF77630.1
4	MT951626.1	A11	China	Asia	UIS31349.1
5	MZ888508.1	GXB08	China	Asia	UZH43805.1
6	GQ166972.1	B-SD-RZ	China	Asia	ACS44345.1
7	JQ315182.1	V478/TW09	Taiwan	Asia	AFV39834.1
8	JQ315173.1	SP-Bursa vac3	Taiwan	Asia	AFV39825.1
9	JQ315171.1	IZO-2512	Taiwan	Asia	AFV39823.1
10	JQ315166.1	CEVA-IBD L	Taiwan	Asia	AFV39818.1
11	JQ315172.1	NABC	Taiwan	Asia	AFV39824.1
12	JQ315170.1	PBG98	Taiwan	Asia	AFV39822.1
13	JQ315168.1	Hipro-CH/80	Taiwan	Asia	AFV39820.1
14	JQ315169.1	228E	Taiwan	Asia	AFV39821.1
15	ON567385.1	IBD/CVAS/6	India	Asia	WEG19346.1
16	OP831905.1	VMC-IBDV-R6	India	Asia	WGW17688.1
17	MK501731.1	IBDV-PSNgp-India	India	Asia	QBO56517.1
18	MN515033.1	SYZA26	Hungary	Europe	QHI42092.1
19	JF965440.1	LD-847–04	Argentina	America	AEL75034.1
20	KC603937.1	IC-IBDV-Br	Brazil	America	AGM16323.1

## Data Availability

The data that support the findings of this study are available from the corresponding author upon reasonable request.
